# Substance Use and Risky Sexual Behavior in the PrEP Outpatient Clinic at the University Hospital of Brasília

**DOI:** 10.3390/tropicalmed8060323

**Published:** 2023-06-16

**Authors:** Alan Rodrigues da Costa, Jônatas Ferreira Barros, Valéria Paes Lima, Camila Magalhães, Hellen Kássia Rezende Silva, Rodolfo Deusdará, Juliana de Souza Lapa

**Affiliations:** Núcleo de Medicina Tropical, Faculdade de Medicina, Campus Universitário Darcy Ribeiro, Universidade de Brasília, Asa Norte, Brasília 70910-900, Brazil

**Keywords:** pre-exposure prophylaxis, recreational drug use, sexually transmitted diseases

## Abstract

(1) Background: To evaluate the epidemiological profile of people who use drugs at the PrEP outpatient clinic of the University Hospital of Brasília; (2) Methods: Cross-sectional study with a review of data from medical records referring to the first medical consultation. The prevalence ratio was calculated using a Poisson regression model with robust variance; (3) Results: A total of 53% of subjects reported drug use in the last 3 months. The unadjusted prevalence ratio of drug use in trans women was PR: 9.0 (95%CI: 1.4–57.5). people who use drugs have a 1.9 times higher prevalence of STI diagnosis, and a 2.4 times higher prevalence of partners compared to non-users; (4) Conclusions: Substance use was associated with a higher STI prevalence ratio and number of sexual partners.

## 1. Introduction

Pre-exposure prophylaxis (PrEP) to the Human Immunodeficiency Virus (HIV) started in Brazil in 2017, aiming to reduce the presence of HIV in high-risk populations through the administration of two antiretroviral medications for HIV-negative patients [1]. PrEP is considered an effective alternative to prevent HIV in patients transmission to patients considered at a high risk of infection, such as gay men and other men who have sex with men (MSM); transgender people; sex workers and serodifferent partners (when one person is infected with HIV and the other is not). There can be a reduction of about 85% in the relative risk of HIV infection when used continuously and under the supervision of health professionals [2]. Recently, the offer of PrEP on-demand was also introduced, that is, the use of the drug PrEP before and after exposure [3]. However, the prophylaxis is not able to prevent the infection of other sexually transmitted infections (STIs) [3].

In Brazil, the Unified Public Healthcare System (Portuguese acronym, SUS) makes PrEP available to people who are 15 years and older, with a bodyweight equal to or greater than 35 kg, who are sexually active and who reports behavior of increased risk of HIV infection. In addition, the assessment of eligibility for PrEP use is carried out during the risk management approach with the individual, observing their sexual practices and partnerships, their social dynamics and the specific contexts associated with an increased risk of infection [4].

Belonging to one of the key population segments—cisgender MSM, transgender people, sex workers and serodifferent partners—is not enough to characterize individuals with frequent exposure to HIV. For this definition, it is necessary to observe the person’s sexual practices and partnerships, their social dynamics and the specific contexts associated with a higher risk of infection, considering other indicators, such as: the repetition of anal or vaginal sexual practices with penetration without the use of condom; the frequency of sexual relations with occasional partners; the number and diversity of sexual partnerships; history of STI episodes; the repeated search for post-exposure prophylaxis (PEP); contexts of sexual relations in exchange for money, valuables, drugs and housing; and sexual practice under the influence of psychoactive drugs (methamphetamines, gamma-hydroxybutyrate (GHB), 3,4-methylenedioxy-methamphetamine (MDMA), cocaine, poppers) with the objective of enhancing or facilitating sexual experiences [4].

In a study of PrEP-eligible individuals in San Diego, California, adults interested in starting PrEP had a higher prevalence of recreational drug use, particularly cocaine, poppers, and ecstasy, compared with those who were not interested in start PrEP [5]. In addition, a Brazilian study of PrEP users showed that 44.3% of its cohort had reported substance use within the 12 months that preceded the study, a much higher frequency than the 3.2% reported by the general population in Brazil [6,7].

Among PrEP users, it is very common to have sex under the influence of psychoactive drugs. Chemsex, chemical sex, is defined as sex under the influence of psychoactive drugs, prolonging and intensifying the pleasure of the sexual act. Main examples are gamma-hydroxybutyric acid (GHB), marketed as “Gisele”, methamphetamines, and nitrate derivatives (known as poppers, a drug that promotes relaxation of anal and oropharyngeal smooth muscles) [8]. The use of parenteral substances, that is substances administered by injection, is referred to, by this population segment, as slamming [9,10]. The use of sexual stimulants for erectile dysfunction without clinical or pathological indication is also considered Chemsex [11]. This practice has been reported to be common in the LGBTQIAP+ population (lesbian, gay, bisexual, trans, queer, intersex, asexual/aromantic/agender and pansexual) and its incidence has increased among men who have sex with men (MSM) in recent years [8]. This increases the risk of STIs infections, as it causes dryness and dehydration of mucous membranes, and may lead to incautious behaviors such as the practice of anal sex without condoms and the practice of group sex without changing condoms as participants have intercourse with multiple partners [8]. In addition, it is known that the use of these drugs causes many adverse effects, such as atrial fibrillation, tachycardia and decreased level of consciousness [12]. However, in a study carried out in the United Kingdom (UK), most Chemsex practitioners did not observe damage or problems in the use of recreational drugs and few practitioners reported harms in the constant use of the practice [13]. In Brazil, there is a growing incidence of MSM who practice Chemsex, with numbers that may surpass European countries [14]. There is an alarming increase in the HIV/AIDS rate (from 24.9 to 28.5 cases/100,000 inhabitants, from 2005 to 2011, and currently, 25.2 cases per 100,000 inhabitants) among MSM in the age group of 18 to 24 years old [8].

The present study aims to evaluate the epidemiological profile of people who use drugs in a cohort of PrEP patients from the outpatient clinic of the University Hospital of Brasília.

## 2. Materials and Methods

### 2.1. Study Design

This work is a cross-sectional study evaluating data from the first consultation of the cohort of subjects taking PrEP medications at the University Hospital of Brasília. It is a review of data from the medical records in the Medication Logistic Control System (Portuguese acronym, SICLOM) of subjects treated at the PrEP outpatient clinic from December 2018 to April 2020. According to the PrEP protocol of the Brazilian Ministry of Health, all subjects must fill out a standardized questionnaire assessing the following dimensions: number of partners, condom use and previous STIs.

The PrEP outpatient clinic at the University Hospital of Brasília began its services in December 2018 and receives subjects who wish to start prophylaxis.

### 2.2. Data Collection

All data were accessed on the SICLOM platform with information regarding PrEP users in the outpatient clinic at the University Hospital of Brasília.

The following socio-demographic data were accessed in the SICLOM AIDS “PrEP User Registration” form: gender, age, education, sexual orientation, skin color.

The following data regarding the three months preceding the first consultation of each PrEP user were accessed through the SICLOM AIDS “PrEP Monitoring” form: Number of partners, condom use, report of sexually transmitted infections (STIs), tests proving the infecction of a new STI and drug use.

The collected data were tabulated in the Research Electronic Data Capture (RedCAP) platform. 

### 2.3. Statistical Analysis

For descriptive analysis, absolute and relative frequencies were calculated for categorical variables. For continuous variables, normality was assessed using the Shapiro–Wilk test. No variable showed normal distribution. Thus, medians and interquartile ranges were calculated for continuous variables.

Two analyses were performed:

(1) The association between socio-demographic factors and drug use

The independent variables were: gender identity (cisgender men, transgender men, cisgender women and transgender women), age, education (≥12 years of study vs. <12 years of study), sexual orientation (heterosexual, homosexual or bisexual), skin color (White, Black, Pardo, Yellow or Indigenous).

The dependent variable was: Drug use, which was defined as having used a substance in the last 3 months.

Unadjusted prevalence ratios were calculated using the Poisson regression model with Robust Variance.

(2) The association between drug use and risky sexual behavior.

The independent variable was: Drug use, which was defined as having used a substance in the last 3 months.

The dependent variables were: Number of partners, condom use, reports of sexually transmitted infections (STIs), tests proving the acquisition of a new STI and drug use.

Unadjusted and adjusted prevalence ratios for gender identity, age, education, and sexual orientation were calculated using the Poisson regression model with Robust Variance.

All the analysis was conducted using STATA version 14 (StataCorp LP, College Station, TX, USA).

## 3. Results

A total of 238 subjects were monitored at the outpatient clinic of the University Hospital of Brasília. Most self-declared as a cisgender man, homosexual and with 12 or more years of education. More than half of the subjects reported drug use and almost half of subjects declared themselves as White. Approximately 20% presented a diagnosis of STI in the last 6 months. It was observed that most subjects were identified as White, male, gay/lesbian, and reported having 12 or more years of education, as can be seen in Table 1.

About half of the subjects (53%) reported using any kind of drug. The most common was marijuana, followed by club drugs, as can be seen in Table 1. Approximately 0.4% reported using crack. A similar pattern has been reported for injectable drugs. Regarding the use of crack and injectable drugs, it was observed that only 0.4% of the subjects.

The unadjusted prevalence ratios for drug use in transgender women and cisgender men, when compared to cisgender women, were PR: 9.0 (95%CI: 1.4–57.5) and PR: 4.5 (95%CI%: 0.7–28.1), respectively. Regarding sexual orientation, the unadjusted prevalence ratios for drug use in homosexual and bisexual people were PR: 3.8 (95%CI: 1.0–14.0) and PR: 3.6 (95%CI: 0.9–14.0), respectively when compared to heterosexual people, as observed in Table 2. 

Using the following outcomes: STI, number of partners, irregular condom use, and use of PEP in the last 6 months, we observed that the prevalence ratios among people who use drugs were, respectively PR: 1.9 (95%CI: 1.2–3.3); PR: 2.4 (95% CI: 1.5–3.8); PR: 1.1 (95%CI: 0.9–1.3) PR: 1.7 (95%CI: 0.9–3.2) compared to people who do not use drugs in the multivariate analysis, as shown in Table 3. 

## 4. Discussion

A high prevalence of psychoactive drug use was observed among patients on PrEP, near eight times more than reported by Brazil’s general population, according to the III National Survey on Drug Use by the Brazilian Population [6]. Another study showed that the use of alcohol and other recreational drugs, such as cocaine and methamphetamines, do not reduce the effectiveness of PrEP, but can impair adherence to the use of the drug [15].

In our cohort, transgender women had a higher prevalence ratio for drug use. The use of illicit substances was significantly high, increasing the prevalence ratio by approximately two times for a greater number of sexual partners and for STIs in in people who use drugs compared people who do not use drugs. A Brazilian study including PrEP users and non-users found that transgender women were 2.44 times more likely than MSM to use substances during sexual intercourse. A higher risk of self-reporting STIs in recent months and more than five sexual partners in the period were also reported in subjects who reported practicing Chemsex [16].

A Canadian study of a cohort of PrEP users following subjects for 24 months found a 32% increased risk of the diagnosis of gonorrhea and chlamydia in subjects practicing Chemsex [17].

Corroborating these findings, a multicenter study carried out in France and Canada evaluated adherence to the PrEP therapeutic scheme and the characteristics associated with habits and risk of infection. It was reported that 29% (95/331) of the participants stated that Chemsex users were twice as likely to use PrEP only the last time they had sex, compared to non-users [18].

Studies have increasingly shown the practice of Chemsex among PrEP users, which draws attention to the need for better assessments of the impact of PrEP use on the sexual behavior of individuals who use drugs and consequent health recommendations for this population [18]. 

A study conducted in the city of Barcelona, Spain, found a prevalence of Chemsex practices among PrEP users higher than described in other studies, as well as other higher-risk sexual practices. The authors reinforced the importance of health professionals increasingly expanding their knowledge of this topic to provide a better support to this population. They also suggested a nonjudgmental and respectful approach to guidance on prevention measures regarding Chemsex, favoring an adequate follow-up, while counseling on drug use and harm-reduction strategies [19].

Strategies that support effective access to PrEP can increase PrEP adherence levels among individuals who practice Chemsex; this result was found by Maxwell, Shahmanesh and Gafos (2022), when assessing PrEP uptake and adherence in the UK. They found that the majority of gay and bisexual men who engaged in Chemsex initiated PrEP in recognition of their potential risk for HIV infection and reported high levels of PrEP adherence. These findings support a growing body of evidence that PrEP is a viable prevention tool for those who engage in Chemsex and that Chemsex does not negatively affect PrEP adherence [20].

Another study reinforced that even in the context of high vulnerability to HIV and other STIs, participants using Chemsex seem to adapt well to the PrEP routine, with good adherence to the pills, and thus remaining protected from HIV infection [21].

Despite these findings, it is important to emphasize that PrEP does not prevent the infection of other STIs, which indicates the need to, when providing health care to this population, always use an interdisciplinary and comprehensive approach that includes information about substances and risk management measures in the context of high-risk sexual practices [22].

In our study, people who use drugs had a two times higher prevalence ratio for a higher number of sexual partners and STIs compared to non-drug users. In a study conducted to investigate STI protection strategies in PrEP users, PrEP users are highly vulnerable to STIs. In addition, individuals who had previous STIs and who report using stimulant drugs or sex work are at higher risk for STI occurrence after starting PrEP [21].

Most STIs are not infections that can occur only once in an individual. On the contrary, reports of people who had several STI episodes throughout their lives are common. One hypothesis for this recurrence would be the behavioral factor, understanding that some individuals, by having a pattern of sexual behavior of exposure to risk throughout their lives, are more predisposed to become infected repeatedly [23].

Since risky sexual behavior can be observed among PrEP users, studies have suggested that making PrEP available is an opportunity to maintain longitudinal follow-up of a population highly vulnerable to STIs. This measure allows for building awareness of the efficacy of PrEP, as well as of the necessary protection against other STIs [21].

The study, which analyzed the databases of the PrEP Demonstration Project in Brazil, identified that STIs were quite frequent among the included participants. Regarding previous STIs found in the study, it was observed that most participants with complete data at study entry had laboratory evidence of having had infections with syphilis, infection with chlamydia trachomatis, neisseria gonorrhoeae or herpes simplex type 2 [21].

Other studies have shown the high prevalence of STIs among PrEP users. In the United Kingdom, the pre-exposure prophylaxis to prevent the acquisition of HIV-1 infection (PROUD) study found that most participants had had an STI diagnosed prior to study entry, and of these most had had a bacterial STI [2]. In contrast, the Ipergay study, conducted in France and Canada, found that a minority of the participants included had had an STI prior to study inclusion [18].

Although some PrEP users may become infected with multiple STIs during their lifetime because they are being followed up for PrEP use, they will have a greater opportunity to identify and treat them quickly. For highly vulnerable individuals, PrEP is being confirmed as the best way to prevent HIV infection. This reinforces the role of health professionals at the PrEP clinic, in the follow-up offered to these people, and makes it even more effective in the control of HIV and other STIs, through guidance in these healthcare settings [21].

A study conducted in Paraná state, Brazil, had the objective of profiling PrEP users assisted by the Counseling and Guidance Center (COA) in Curitiba. The findings showed a higher incidence of syphilis in individuals with a higher number of sexual partners after the implementation of PrEP in the study population, demonstrating that, during clinical follow-up in PrEP users, adverse events and the occurrence of other STIs can be effectively monitored [24].

The orientation of the population vulnerable to HIV infection and other STIs through pre-exposure prophylaxis in outpatient clinics is an important health and public policy action, which complements other actions such as guidance on sexual education, encouraging the use of condoms and the testing and treatment of STIs [24]. Our study did not find any statistically significant values in the prevalence ratios of drug use associated with socio-demographic data such as color or education. This can be explained by the homogeneity of the patients, because 82% self-declared as White or Pardo and 92% had 12 or more years of education. PrEP subjects at the University Hospital of Brasília are mostly white, male, gay/lesbian, and with more than 12 years of schooling. According to a recent survey on the heterogeneity of men who have sex with men (MSM) in Brasília, Brazil’s capital, is the city with the highest income and education levels, corroborating the characteristics of the subjects observed in our outpatient clinic [25].

Regarding race/color, another study also found a majority of users reported as White and strongly associated with higher education levels (*p* < 0.001). Other studies pointed to an association of that profile with better adherence to PrEP. As a matter of fact, there is a hypothesis that the tendency for worse adherence among non-White users is due to other social determinants of the health-disease process, such as structural racism and poor access to healthcare [26].

A study carried out in the first six months of PrEP provision in the statf of Paraná, Brazil, found a predominance of males (91.4%), identified as cis men (88.6%), homosexuals (78.0%), between 20 and 39 years old (83.5%), White skin color (71.8%) and with 12 years or more of education (74.9%) among individuals assisted in the state’s specialized HIV/AIDS care services [27].

Another study conducted in the state of Alagoas, Brazil, found that PrEP users in that state are predominantly men-cis, Pardo, aged 30 years or older, homosexual/gay/lesbian, who perform anal sexual intercourse and have 12 or more years of schooling. It draws attention that in both studies the profile found was of male users, homosexuals, with 12 years of schooling [28].

The high level of education among the individuals found in this and other studies reinforces the hypothesis that a less educated and more marginalized population is less likely to use PrEP due to the lack of information regarding prevention and follow-up, and due to barriers they may face in order to access healthcare [29].

In our study, no significant difference was observed in the prevalence ratio of the variables “irregular condom use” and “PEP use in the last six months” after adjusting for gender identity, age, education, and sexual orientation. The question about the use of condoms after the introduction of PrEP is still uncertain, given that some studies reported that after the start of treatment, there was a reduction in use, and other studies have already found an increase in the practice of protection [30,31].

Although the use of condoms is the best way to avoid STIs, only half of this population uses them, including in sexual relations with casual partners, so the use of PrEP, in situations of high vulnerability, can be an important tool to protect against HIV infection. Furthermore, consistent studies have found no association between PrEP use and changes in sexual risk behavior. In this way, PrEP may be the appropriate method for preventing HIV infection in people who have unprotected sex and in those with difficulties in negotiating other preventive methods, subject to sexual violence, sex workers or who perform sexual practices involving alcohol and other drugs [32,33].

Our study has some limitations. It was not possible to evaluate the practice of Chemsex because this question was not included in the first consultation form. As it is a cross-sectional study, it is subject to selection/survival bias and temporal bias. It should be noted that this is a study with a small convenience sample, which does not allow data generalization. The fact that this is a homogeneous sample from a socio-demographic point of view may have influenced the fact that these aspects were not associated with drug use. However, this is a recurring problem in studies of PrEP users. It is important to note that the data were self-reported, which could lead to information bias, especially when reporting data on drug use, number of partners and irregular use of condoms due to social-desirability bias. Although these study limitations, our data provide valuable insights into important aspects regarding the profile of PrEP users subjects in key populations. 

Important public health policies, such as health promotion and HIV prevention with access to PEP and PrEP in key populations, may reduce risky behavioral measures, such as Chemsex, leading to a reduction in new cases of STI [34]. This is particularly true considering the most vulnerable people such as transgender women [35]. Furthermore, the greater success of PrEP as a public health policy depends on ensuring that services are culturally diverse and discrimination-free. It also depends on the intensification of community interventions (population embracement), including social networks, which help reduce inequities in access to services and PrEP [30,34]. The ample access to PrEP-HIV treatment is an important public health strategy for local and national HIV prevention, as it helps decrease the number of new infections.

## 5. Conclusions

Male, White and homosexual people and people with more than 12 years of schooling were predominant among people who use drugs in this cohort of subjects on PrEP at the outpatient clinic of the University Hospital of Brasilia.

Drug use in PrEP users was associated with a higher prevalence ratio of STI diagnoses and a greater number of partners compared to non-drug users in the PrEP outpatient clinic at the University Hospital of Brasília in the period evaluated. In addition, a high prevalence of drug use in the last 6 months was observed in the PrEP user’s population (53%).

Therefore, this subgroup is very vulnerable to STIs infections. It requires a more focused approach to counter high risk behavior, such as the expansion of services through inclusive policies that can ensure their rights and access to quality health care. New prospective studies that assess the relationship between drug use during sex are needed to better estimate this practice in Brazil.

## Figures and Tables

**Table 1:** Characteristics of the 238 subjects monitored at the Pre-Exposure Prophylaxis Outpatient Clinic of the University Hospital of Brasília from 2018 to 2020.

Variables	n (%)
Gender identity	
Cisgender men	216 (94.3%)
Cisgender women	9 (3.9%)
Transgender women	3 (1.3%)
Transgender men	1 (0.43%)
Age (years) ^1^	31 (26–45)
Race/ethnicity	
White	106 (45.5%)
Pardo	86 (36.9%)
Black	37 (15.8%)
Indigenous	2 (0.85%)
Yellow	2 (0.85%)
Education	
≥12 years	214 (91.8%)
<12 years	19 (8.2%)
Sexual orientation	
Homosexual	188 (81%)
Bisexual	29 (12.5%)
Heterosexual	15 (6.5%)
Sex Worker	22 (9.2%)
Taking any kind of drug	126 (53%)
Marijuana	73 (30.6%)
Club drugs	62 (26%)
Cocaine	35 (14.7%)
Poppers	33 (13.8%)
Solvent	11 (4.6%)
Crack	1 (0.42%)
Injectable drugs	1 (0.42%)
STI diagnosis in the last 6 months	
Any STI ^2^	49 (21%)
Syphilis	16 (6.7%)
Gonococcus and chlamydia	11 (4.6%)

^1^ Median and interquartile range. ^2^ Including syphilis, gonococcus and Chlamydia, herpes lesions, HPV, other types of urethral discharge and lesions diagnosed as STIs.

**Table 2:** Unadjusted prevalence ratio (PR) between associated factors and drug use in 238 subjects followed at the Pre-Exposure Prophylaxis Outpatient Clinic of the University Hospital of Brasília from 2018 to 2020.

	Drug Use
	Unadjusted PR (IC 95%)
Gender identity	
Cisgender women	(Reference)
Cisgender men	4.5 (0.7–28.1)
Transgender women	9.0 (1.4–57.5)
Race/ethnicity	
White	(Reference)
Pardo	1.0 (0.7–1.6)
Black	1.1 (0.8–1.6)
Indigenous	1.0 (0.3–4.2)
Yellow	1.0 (0.3–4.2)
Education	
≥12 years	(Reference)
<12 years	0.9 (0.5–1.5)
Sexual orientation	
Heterosexual	(Reference)
Homosexual	3.8 (1.0–14.0)
Bisexual	3.6 (0.9–14.0)
Prostitution	1.3 (0.9–1.8)

**Table 3:** Unadjusted and adjusted prevalence ratios (PR) ^1^ (95% confidence intervals) between drug use and sexually transmitted infections (STIs) and factors associated with contracting STIs in subjects followed at the Pre-Exposure Prophylaxis Outpatient Clinic of the University Hospital of Brasilia from 2018 to 2020.

	STIs		No. of Partners		Irregular Condom Use		Taking PEP in the Last 6 Months	
	Unadjusted PR	Adjusted PR ^1^	Unadjusted PR	Adjusted PR ^1^	Unadjusted PR	Adjusted PR ^1^	Unadjusted PR	Adjusted PR ^1^
People who do not use drugs	Reference		Reference		Reference		Reference	
People who use drugs	2.2 (1.3–3.8)	1.9 (1.2–3.3)	2.5 (1.3–4.7)	2.4 (1.5–3.8)	1.2 (1.00–1.4)	1.1 (0.9–1.3)	1.7 (1.00–3.00)	1.7 (0.9–3.2)

^1^ Adjusted for sex, age, educational status, sexual orientation.

## Data Availability

The data presented in this study are available upon request from the corresponding author.
